# Survival and prognostic factors in chondrosarcoma: Results in 115 patients with long-term follow-up

**DOI:** 10.3109/17453674.2011.636668

**Published:** 2011-11-25

**Authors:** Dimosthenis Andreou, Sebastian Ruppin, Sebastian Fehlberg, Daniel Pink, Mathias Werner, Per-Ulf Tunn

**Affiliations:** ^1^Department of Orthopedic Oncology, Sarcoma Center Berlin-Brandenburg, Helios Klinikum Berlin-Buch, Berlin; ^2^Department of Hematology, Oncology and Palliative Care, Sarcoma Center Berlin-Brandenburg, Helios Klinikum Bad Saarow, Bad Saarow; ^3^Department of Pathology, Sarcoma Center Berlin-Brandenburg, Helios Klinikum Emil von Behring, Berlin, Germany

## Abstract

**Background and purpose:**

There have been few long-term studies on the outcome of chondrosarcoma and the findings regarding prognostic factors are controversial. We examined a homogeneous group of patients with primary central chondrosarcoma of bone who were treated according to a uniform surgical protocol at our institution, in order to determine the factors that influence survival and identify potential improvements to our therapeutic algorithm.

**Patients and methods:**

We performed a retrospective analysis of 115 patients with primary central chondrosarcoma of bone who presented with localized disease and who had a minimum follow-up of 5 years after diagnosis. 68 tumors were localized in the extremities and 47 in the axial skeleton or pelvis. 59 patients had a high-grade (II and III) and 56 a low-grade (I) tumor. 94 patients underwent surgical resection with adequate (wide or radical) margins, while 21 patients had inadequate (marginal or intralesional) margins.

**Results:**

Tumor grade and localization were found to be statistically significant independent predictors of disease-related deaths in multivariate analysis. The quality of surgical margins did not influence survival. The AJCC staging system was able to predict prognosis in patients with chondrosarcoma of the extremities, but not in those with tumors of the axial skeleton and pelvis. Long-term survival after secondary metastatic disease was only observed when metastases were resected with wide margins. Patients with metastases who received further treatment with conventional chemotherapy, radiotherapy, and/or further surgery had significantly better survival compared to those who received best supportive care.

**Interpretation:**

The outcome in patients with primary central chondrosarcoma of bone who present with localized disease is mostly affected by tumor-related parameters.

Chondrosarcoma is the second most common primary malignant solid tumor of bone, and accounts for approximately 25% of all bone sarcomas (Bertoni et al. 2002). It is largely considered to be resistant to conventional chemotherapy and radiotherapy (Healey and Lane 1986, Campanacci 1999, Gelderbloom et al. 2008). As such, surgical resection has been the cornerstone of treatment for over 50 years (Dahlin and Henderson 1956, Healey and Lane 1986, Gelderbloom et al. 2008). However, in recent years several novel therapeutic approaches have been evaluated in experimental studies (Morioka et al. 2003, Gouin et al. 2006, Klenke et al. 2007, Delaney et al. 2009, Schrage et al. 2009, 2010).

There is no consensus on prognostic factors to determine which patients have a higher risk of treatment failure and disease-related deaths, although several papers have addressed this issue (Evans et al. 1977, Pritchard et al. 1980, Gitelis et al. 1981, Björnsson et al. 1998, Lee et al. 1999, Rizzo et al. 2001, Fiorenza et al. 2002). One reason may be that most studies have included patients treated over several decades, with no account for the different surgical criteria, indications, and methods applied over the years. Furthermore, most studies have included patients with short follow-up, despite the fact that a high rate of late recurrence and metastasis has been reported for chondrosarcoma patients compared to those with other primary bone sarcomas (Evans et al. 1977, Pritchard et al. 1980), as well as patients with rare histopathological subtypes that have a distinct biologic behavior (Lee et al. 1999, Bertoni et al. 2002, Gelderbloom et al. 2008) such as dedifferentiated chondrosarcoma, mesenchymal chondrosarcoma, and clear cell chondrosarcoma, thus reducing the validity of the results.

The purpose of this long-term retrospective study was to examine a group of patients with primary central chondrosarcoma of bone who presented with localized disease and were treated with a uniform surgical protocol at our institution, in order to determine the factors that influence overall and event-free survival. We further aimed at identifying potential improvements to our therapeutic algorithm.

## Patients and methods

146 patients with primary central chondrosarcoma of bone presenting with localized disease were treated at our sarcoma department between 1982 and 2004. 31 patients were excluded from this analysis—6 patients who were primarily treated with palliative intent and 25 patients with follow-up of less than 5 years after diagnosis—leaving 115 patients for this study. At hospital admission, all patients had signed a consent form allowing the use of anonymized information for research purposes.

There were 70 male and 45 female patients. The mean age at presentation was 47 (14–79) years and 81 patients were older than 40 years (Table 1). The mean follow-up period for survivors was 12 (5–24) years. Follow-up data were obtained at our outpatient clinic or by telephone calls to referring physicians.

**Table 1.** Distribution of patient age

Patient age	n	%
14–20	9	8
21–30	8	7
31–40	17	15
41–50	26	22
51–60	33	29
61–70	16	14
71–79	6	5

48 tumors were located in the lower extremity, 42 in the pelvic girdle, 20 in the upper extremity or shoulder girdle, and 5 in the axial skeleton. 8 patients presented with a pathological fracture in the lower (n = 5) or upper (n = 3) extremity. Tumor volume was assessed by the pathologist during examination of the surgical specimen. Recorded for 93 patients, it had a mean of 396 (1–3,827) cm^3^.

56 patients (49%) were diagnosed with grade I tumors, 41 patients (36%) with grade II, and 18 patients (15%) with grade III tumors. The grading was based on the system proposed by Evans et al. (1977). Cartilage tumors of borderline malignancy were not included in this series. According to the American Joint Committee on Cancer (AJCC) staging system (Greene et al. 2002), 31 patients had stage IA tumors (27%), 23 patients stage IB (20%), 10 patients stage IIA (9%), and 51 patients stage IIB tumors (44%). For the statistical analysis, grade II and grade III tumors were classified as high-grade, while grade I tumors were classified as low-grade.

All patients underwent surgical treatment of the primary tumor. Customized megaprostheses became available at our institution in 1982. Since then, our surgical protocol has undergone only minor alterations, consistently involving the curettage of small, low-grade tumors of the extremities without infiltration of the soft tissue, and a planned wide resection followed by a biological or endoprosthetic reconstruction—when necessary—for all other tumors. Amputations were performed when limb-sparing procedures with wide surgical margins and preservation of a functional limb were deemed impossible, mainly due to tumor infiltration of neurovascular structures or extensive soft tissue infiltration, unless the patient declined to undergo a mutilating procedure.

Surgical margins were divided into intralesional, marginal, wide and radical, according to the classification of Enneking. For the purposes of this analysis, intralesional and marginal margins, documented in 21 patients (18%), were characterized as inadequate. Among them were 11 patients in whom a wide resection was initially planned; 4 of these patients underwent a wide re-resection, while 1 patient received adjuvant radiation therapy with 60 Gy. Wide and radical margins, documented in 94 patients (82%), were characterized as adequate surgical margins. 77 patients with tumors of the extremities or pelvic girdle underwent limb-sparing surgery, while 33 patients were amputated.

A local recurrence developed in 38 patients (33%) after a mean of 21 (2–96) months. In 2 of these patients, the tumor had dedifferentiated. Distant metastasis developed in 30 patients (26%) after a mean of 27 (2–141) months. 19 patients (17%) developed both a local recurrence and distant metastases at some stage of their illness.

### Statistics

Survival analysis was performed using the Kaplan-Meier method. Overall survival was calculated from the date of diagnostic biopsy until death related to disease or treatment, and event-free survival from the date of tumor resection until disease recurrence or death. Survival curves were compared using the log-rank test. Taking into account the different challenges in surgical treatment of tumors of the axial skeleton and pelvic girdle compared to tumors of the extremities, as well as the differences in the clinical course of low- and high-grade tumors, overall and event-free survival were calculated separately for these groups. We performed a multivariate analysis with the Cox proportional hazards model to identify independent predictors of survival in the entire group. The variables entered into this model were the ones previously examined in the literature, in order to permit a direct and consistent comparison of our results with the results of earlier studies. The proportional hazards assumption was checked by plotting the logarithm of the cumulative hazards functions for each covariate, with parallel curves supporting the proportional hazards assumption. The continuous covariate age was categorized with a cutoff at 40 years. All statistical analyses were carried out using the SPSS software version 16.0 (SPSS, Inc., Chicago, IL). All tests were 2-sided. A p-value of less than 0.05 was considered significant.

## Results

**Figure 1.**
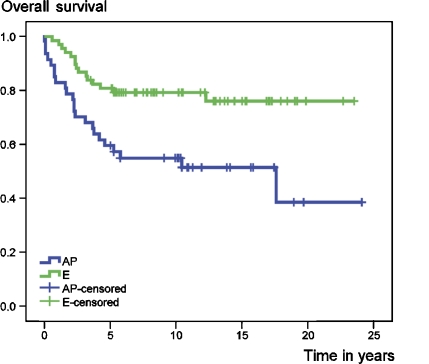
The impact of tumor location on overall survival.

At the time of this analysis, 73 patients were alive with no evidence of disease. 32 patients had died from disease, 6 patients from treatment-related complications and 4 patients from other causes. Overall survival rates of the entire group at 5 and 10 years were 72% and 69%, respectively. Event-free survival at 5 and 10 years amounted to 57% and 53%, respectively. Both overall (Figure 1) and event-free survival were statistically significantly better for patients with chondrosarcomas of the extremities (E group) than for those with tumors of the axial skeleton and pelvic girdle (AP group) (Table 2).

**Table 2.** Univariate analysis of overall and event-free survival

		Overall survival (%)			Event-free survival (%)		
	Patients	5-year	10-year	p-value **^a^**	5-year	10-year	p-value **^a^**
Entire group	115	72	69		57	53	
AP **^b^**	47	60	55	0.002	40	38	0.004
E **^c^**	68	81	79		68	63	
Low-grade	56	89	89	< 0.001	73	68	< 0.001
High-grade	59	56	50		41	39	
Age							
≤ 40 years	34	85	82	0.04	62	55	0.6
> 40 years	81	67	64		54	52	
Sex							
Male	70	73	68	0.6	56	52	0.9
Female	45	71	71		58	54	
Tumor volume in cm^3^							
0–100	36	92	89	< 0.001	81	76	0.007
100–200	18	50	50		44	44	
100–200	18	50	50	1.0	44	44	1.0
> 200	39	61	58		46	43	
Local recurrence							
No	77	83	83	< 0.001			
Yes	38	47	42				
AP – No	27	74	74	0.005			
AP – Yes	20	40	29				
E – No	50	90	88	< 0.001			
E – Yes	18	56	56				
Low-grade – No	41	95	95	0.008			
Low-grade – Yes	15	73	73				
High-grade – No	36	72	69	< 0.001			
High-grade – Yes	23	30	21				
Distant metastasis							
No	85	89	89	< 0.001			
Yes	30	23	13				
AP – No	29	76	76	< 0.001			
AP – Yes	18	33	22				
E – No	56	96	96	< 0.001			
E – Yes	12	8	0				
Low-grade – No	53	94	94	< 0.001			
Low-grade – Yes	3	0	0				
High-grade – No	32	81	81	< 0.001			
High-grade – Yes	27	26	15				
Tumor grade							
G1	56	89	89	< 0.001	73	68	< 0.001
G2	41	63	58		49	49	
G3	18	39	33		22	15	
AP – G1	18	72	72	0.04	56	56	0.001
AP – G2	19	58	53		42	42	
AP – G3	10	40	30		10	0	
E – G1	38	97	97	< 0.001	82	74	0.02
E – G2	22	68	63		54	54	
E – G3	8	38	38		38	38	
Surgical margins							
Inadequate	21	71	67	0.9	48	33	0.2
Adequate	94	72	70		59	57	
AP – Inadequate	6	33	17	0.1	0	0	0.08
AP – Adequate	41	63	61		46	44	
E – Inadequate	15	87	87	0.4	67	46	0.4
E – Adequate	53	79	77		68	68	
Low-grade – Inadequate	13	85	85	0.7	69	43	0.2
Low-grade – Adequate	43	91	91		74	74	
High-grade – Inadequate	8	50	38	0.8	13	13	0.3
High-grade – Adequate	51	57	52		45	43	
Type of surgery							
Low-grade – ablative	6	100	100	0.7	67	67	0.9
Low-grade – limb-sparing	46	89	89		74	68	
High-grade – ablative	27	52	43	0.1	37	32	0.4
High-grade – limb-sparing	31	58	58		45	45	
Pathological fracture							
E – No	60	85	85	0.002	72	66	0.1
E – Yes	8	50	38		38	38	
Lower extremity – No	43	86	86	< 0.001	67	67	0.07
Lower extremity – Yes	5	40	20		40	20	
Upper extremity – No	17	82	82	0.4	82	64	0.7
Upper extremity – Yes	3	67	67		67	67	
Tumor stage (AJCC)							
Ia	31	90	90	< 0.001	81	72	0.004
Ib	23	87	87		65	65	
IIa	10	50	50		50	50	
IIb	51	59	53		39	37	
AP – Ia	7	57	57	0.1	57	57	0.3
AP – Ib	11	82	82		55	55	
AP – IIa	5	20	20		20	20	
AP – IIb	24	58	50		33	29	
E – Ia	24	100	100	0.001	88	76	0.02
E – Ib	12	92	92		75	75	
E – IIa	5	80	80		80	80	
E – IIb	27	59	55		44	44	

**^a^** (log-rank)

**^b^** AP: chondrosarcoma of the axial skeleton and pelvic girdle;

**^c^** E: chondrosarcoma of the extremity.

No statistically significant difference was detected in the survival of male and female patients (Table 2). On the other hand, age at diagnosis had a statistically significant impact on overall survival. With a cutoff at 40 years, younger patients faired better than older ones (Table 2). Patients with a tumor volume lower than 100 cm^3^ had a statistically significantly better overall and event-free survival than those with a tumor volume of between 100 cm^3^ and 200 cm^3^. The influence of volume on survival was diminished in tumors greater than 100 cm^3^ (Table 2).

A high tumor grade had a statistically significant negative impact on both overall and event-free survival. The influence on overall survival was far more pronounced in the E group than in the AP group (Table 2), probably due to the worse survival of patients with low-grade tumors in the axial skeleton or pelvis compared to the survival of those with low-grade tumors of the extremities. Regarding event-free survival, only 1 of 10 patients with a grade-III tumor in the AP group remained event-free at 5 years and none remained event-free at 10 years (Table 2).

**Figure 2.**
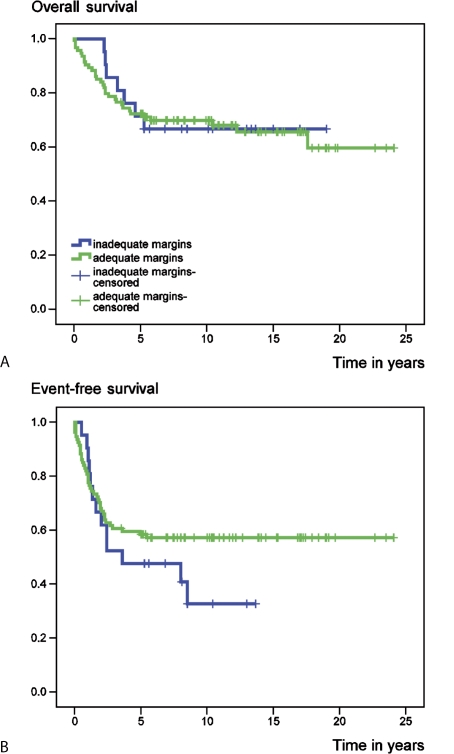
Overall survival (A) and event-free survival (B), according to surgical margins.

The quality of surgical margins did not significantly affect overall or event-free survival (Figure 2), regardless of tumor grade or localization (Table 2). However, subgroup analysis revealed an improved, but not statistically significant, event-free and overall survival for patients with adequate margins in the AP group. Survival was similar between limb-sparing and ablative procedures (Table 2). The presence of a pathological fracture at diagnosis led to a statistically significant decline in overall survival in the entire E group; however, subgroup analysis showed the significance to be restricted to patients with tumors of the lower extremity (Table 2).

**Figure 3.**
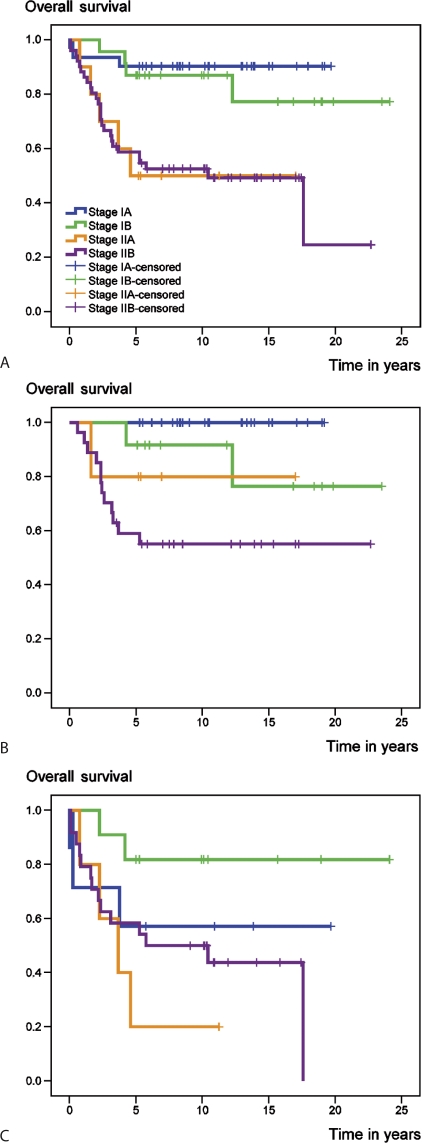
Overall survival in the entire group (A), the E group (B), and the AP group (C) according to the AJCC staging system.

The AJCC staging system correlated both with overall (Figure 3A) and with event-free survival in the entire group (Table 2). This correlation was very strong only in patients with chondrosarcoma of the extremities (Figure 3B). The staging system failed to allow accurate prediction of prognosis in patients with chondrosarcoma of the axial skeleton or pelvis (Figure 3C and Table 2).

**Figure 4.**
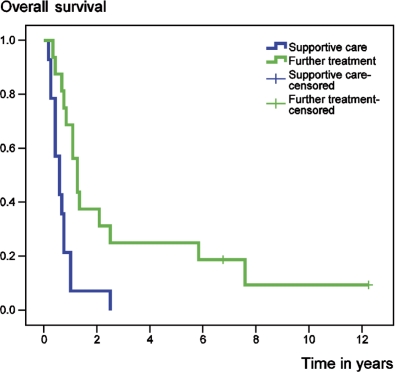
Overall survival following the development of metastatic disease, according to further treatment.

Both the development of local recurrence and the development of distant metastases led to a significant decline in overall survival, regardless of tumor grade or localization (Table 2). A local recurrence preceded the diagnosis of metastatic disease in 10 of 30 patients. Metastatic disease was documented in 3 patients with initially grade I tumors initially, all of whom had previously developed a high-grade local recurrence without dedifferentiation and all of whom died of their disease. Further analysis of the 30 patients with metastases revealed that long-term survival was observed only in patients who had undergone surgical resection of all metastases with wide margins. Patients who received further treatment with conventional multidisciplinary treatment plans, including standard systemic chemotherapy, radiotherapy, and/or further surgery had better survival than those who received best supportive care (p = 0.001) (Figure 4).

In an attempt to identify independent predictors of survival at the time of diagnosis, tumor localization and grade, patient age, sex, and the quality of surgical margins were submitted to multivariate analysis with the Cox proportional hazards regression model. The presence of a pathological fracture at diagnosis was excluded from the multivariate analysis, since this factor appeared only in the E group. Significant independent predictors of death were high grade (relative risk (RR) = 5, 95% CI: 2–12; p < 0.001) and a tumor localization in the axial skeleton and pelvis (RR = 2, 95% CI: 1–4; p = 0.04).

## Discussion

One of the most interesting findings in our series concerns the quality of surgical margins. No effect could be shown on overall survival, both in univariate and in multivariate analysis, although a strong trend of improved survival was found in the AP group for adequate margins. At first, this finding seems to contradict previous studies, which demonstrated that inadequate surgical margins were associated with poorer prognosis (Gitelis et al. 1981, Lee et al. 1999, Rizzo et al. 2001, Fiorenza et al. 2002). However, a systematic review of the literature revealed only 2 studies that included a multivariate analysis for factors affecting survival (Lee et al. 1999, Fiorenza et al. 2002). Inadequate surgical margins were shown to have only minimal influence on overall survival in the first study and no influence in the second. As both studies found that the development of local recurrence was associated with worse overall survival and that inadequate surgical margins led to a higher rate of local recurrence (findings we were able to confirm in our data), the authors postulated that there may be an association between inadequate margins and worse overall survival (Lee et al. 1999, Fiorenza et al. 2002). In light of our results, we believe this is not the case. It has long been shown in soft tissue sarcoma that studies addressing the issue of diminishing local recurrence in a prospective, randomized fashion—such as was done with radiotherapy—did not lead to any survival benefit (Brennan 2007), although it should be noted that no analogous studies have been conducted in chondrosarcoma patients. On these grounds, it has been hypothesized that not all local recurrences are the same (Brennan 2007) and that the development of local recurrence after adequate treatment might be a marker of an aggressive tumor that is more likely to metastasize (Brennan 1997, Gronchi et al. 2007). This, however, may not be the case for local recurrences after intralesional or marginal resections, at least in soft tissue sarcomas (Brennan 2007).

Given that the development and subsequent treatment of a local recurrence can lead to increased morbidity and compromise functional outcome, we do not suggest that these results can justify inadequate surgery of otherwise resectable tumors—with the exception of small, low-grade tumors of the extremities without infiltration of the soft tissue. However, we do think that, in light of these findings, the role of ablative surgery in patients with locally advanced tumors of the extremities for which a limb-sparing resection with wide margins is deemed risky should be examined further. This especially so in patients who refuse amputation or in patients for whom a secondary amputation is recommended after inadequate resection of the primary tumor, as there was no correlation between type of surgery and outcome in our patient cohort.

Another surprising finding was that the AJCC staging system did not correlate with oncological outcome in patients with tumors of the axial skeleton or pelvis. To our knowledge, this has not been reported previously. The reasons for this are unclear. The retrospective nature of our study did not allow an evaluation of the Musculoskeletal Tumor Society (MSTS) staging system, as accurate data regarding tumor compartmentalization were missing in many cases. Heck et al. (2003) found a similar prognostic significance of the AJCC and the MSTS staging systems in patients with primary malignant bone tumors, but subgroup analysis according to tumor localization was not performed. We believe that further studies including all primary bone sarcomas of the pelvis and axial skeleton are warranted; if our results are confirmed, the development of a separate staging system for these tumors would be justified. A possible alternative to tumor size might be tumor volume, with a cutoff at 100 cm^3^, as it was shown to be an important predictor of overall and event-free survival. With volumetric data missing in 7 out of 47 patients in the AP group, such an alternative staging system correlated better with overall and event-free survival in our series—but also failed to reach statistical significance (data not shown).

A dismal prognosis after development of secondary metastatic disease has been reported in many studies (Björnsson et al. 1998, Lee et al. 1999, Rizzo et al. 2001, Fiorenza et al. 2002). In our series, long-term survival was possible only for patients who underwent surgical resection with adequate margins, as has already been described for osteosarcoma patients (Kempf-Bielack et al. 2005). Moreover, further treatment prolonged survival of patients with metastasis compared to best supportive care. However, the small patient sample, the variety of treatment protocols used for metastatic disease at our institution over the years, and the retrospective design of the study preclude any definitive conclusions.

3 of the 56 patients with initially grade I chondrosarcoma developed metastasis and died of their disease within 5 years. Several series examining the course of grade I chondrosarcoma have reported similar results, i.e. a low risk of metastatic disease but high mortality rates after metastasis ranging from 70% to 100% (Leerapun et al. 2007, Schwab et al. 2007, Streitbürger et al. 2009). It appears that the development of metastasis in these patients is suggestive of a highly aggressive disease phenotype.

The presence of a pathological fracture at diagnosis led to a statistically significant decrease in overall survival in patients with chondrosarcoma of the lower extremity, but not in those with tumors of the upper extremity. Lee et al. (1999) found similar survival rates in patients with or without pathological fracture, while Bramer et al. (2007) found a statistically significant decrease in univariate analysis, but not in multivariate analysis. Neither study distinguished between tumors of the lower and the upper extremity; it would be interesting to see whether tumor localization influences the prognostic impact of a pathological fracture in a larger series.

In conclusion, the outcome of patients with primary central chondrosarcoma of bone who present with localized disease is mostly affected by tumor- and patient-related parameters. There appear to be subgroups of patients who might benefit from modifications in the standard practice, including those who have traditionally been considered as candidates for ablative surgery, as well as those with distant metastasis who are unwilling or unable to participate in phase II clinical trials. The AJCC staging system does not appear to accurately predict the risk of disease-specific mortality for all subgroups of patients.
